# Enhanced proteasomal activity is essential for long term survival and recurrence of innately radiation resistant residual glioblastoma cells

**DOI:** 10.18632/oncotarget.25351

**Published:** 2018-06-12

**Authors:** Jacinth Rajendra, Keshava K. Datta, Sheikh Burhan Ud Din Farooqee, Rahul Thorat, Kiran Kumar, Nilesh Gardi, Ekjot Kaur, Jyothi Nair, Sameer Salunkhe, Ketaki Patkar, Sanket Desai, Jayant Sastri Goda, Aliasgar Moiyadi, Amit Dutt, Prasanna Venkatraman, Harsha Gowda, Shilpee Dutt

**Affiliations:** ^1^ Shilpee Dutt Laboratory, Tata Memorial Centre, Advanced Centre for Treatment, Research and Education in Cancer (ACTREC), Kharghar, Navi Mumbai, India; ^2^ Institute of Bioinformatics, International Technology Park, Bangalore, India; ^3^ Advanced Centre for Treatment, Research and Education in Cancer (ACTREC), Tata Memorial Centre (TMC), Kharghar, Navi Mumbai, India; ^4^ Integrated Genomics Laboratory, Advanced Centre for Treatment, Research and Education in Cancer, Tata Memorial Centre, Navi Mumbai, Maharashtra, India; ^5^ Laboratory Animal Facility, Advanced Centre for Treatment, Research and Education in Cancer (ACTREC), Tata Memorial Centre (TMC), Kharghar, Navi Mumbai, India; ^6^ Department of neurosurgery Tata Memorial Centre, Advanced Centre for Treatment, Research and Education in Cancer, Navi Mumbai, India; ^7^ Homi Bhabha National Institute, Training School Complex, Anushakti Nagar, Mumbai, India; ^8^ Department of Radiation Oncology, Tata Memorial Centre, Advanced Centre for Treatment, Research and Education in Cancer, Navi Mumbai, India

**Keywords:** glioblastoma, radio-resistant cells, recurrence, proteomic analysis, proteasomes

## Abstract

Therapy resistance and recurrence in Glioblastoma is due to the presence of residual radiation resistant cells. However, because of their inaccessibility from patient biopsies, the molecular mechanisms driving their survival remain unexplored. Residual Radiation Resistant (RR) and Relapse (R) cells were captured using cellular radiation resistant model generated from patient derived primary cultures and cell lines. iTRAQ based quantitative proteomics was performed to identify pathways unique to RR cells followed by *in vitro* and *in vivo* experiments showing their role in radio-resistance. 2720 proteins were identified across Parent (P), RR and R population with 824 and 874 differential proteins in RR and R cells. Unsupervised clustering showed proteasome pathway as the most significantly deregulated pathway in RR cells. Concordantly, the RR cells displayed enhanced expression and activity of proteasome subunits, which triggered NFkB signalling. Pharmacological inhibition of proteasome activity led to impeded NFkB transcriptional activity, radio-sensitization of RR cells *in vitro*, and significantly reduced capacity to form orthotopic tumours *in vivo*. We demonstrate that combination of proteasome inhibitor with radio-therapy abolish the inaccessible residual resistant cells thereby preventing GBM recurrence. Furthermore, we identified first proteomic signature of RR cells that can be exploited for GBM therapeutics.

## INTRODUCTION

Glioblastoma is the most common and lethal primary brain tumour. Despite the multimodal therapy, tumour recurrence is major challenge in glioblastoma with patient survival less than 6 months post recurrence [1–4]. Recurrence in GBM is attributed to a subpopulation of cells that survive initial therapies and cause tumour re-growth [5, 6]. However, targeting residual resistant cells of glioma is challenging since they are invisible in MRIs post initial treatment and they are inaccessible from the patient biopsies for biological studies [7, 8]. We have previously reported development of a cellular model of radiation resistance using primary cultures from patient samples, which recapitulate the clinical scenario of resistance and enable us to capture residual radiation resistant (RR) cells [9] and understand their molecular mechanism of survival.

Since proteins are the ultimate biological effectors of the cells, in this study we have analyzed the total proteome of residual resistant cells of glioma [10–13]. Till date majority of proteomics studies in glioblastoma have focused on identification of differential proteins amongst different GBM cell lines, patient samples or within the same tumour to investigate the heterogeneity of glioblastoma, mechanism of chemoresistance and identification of diagnostic biomarkers [14–26]. However, none of these studies could identify survival mechanism of innately resistant cells due to their unavailability. This is the first report to identify the proteomic signature of residual resistant and the relapse cells of glioblastoma from cellular model. Data revealed a unique proteomic signature of RR and R cells with utmost clustering of deregulated genes uniquely in the RR cells. Contrary to previous reports which have shown a decrease in proteasome activity in radio resistant cells [27, 28], our data reveals that innately radio resistant GBM cells harbour increased expression of proteasomal subunits, enhanced proteasome activity and increased levels of proteasome substrate p-NFkB and concordant increase of NFkB target genes. We demonstrate pharmacological inhibition of proteasomal activity reduces NFkB transcriptional activity and radio sensitizes RR cells. Furthermore absence of proteasome activity in RR cells also significantly decreases their ability to form tumours *in vivo.* Together, our proteomics data has delineated proteasomal pathway as one of the plausible targetable mechanisms that significantly contribute to the survival of innate radiation residual cells via the NFkB signalling cascade.

## RESULTS

### Capturing innate radiation resistant (RR) and Relapse (R) cells from *in vitro* radiation resistant model

**Figure 1:**
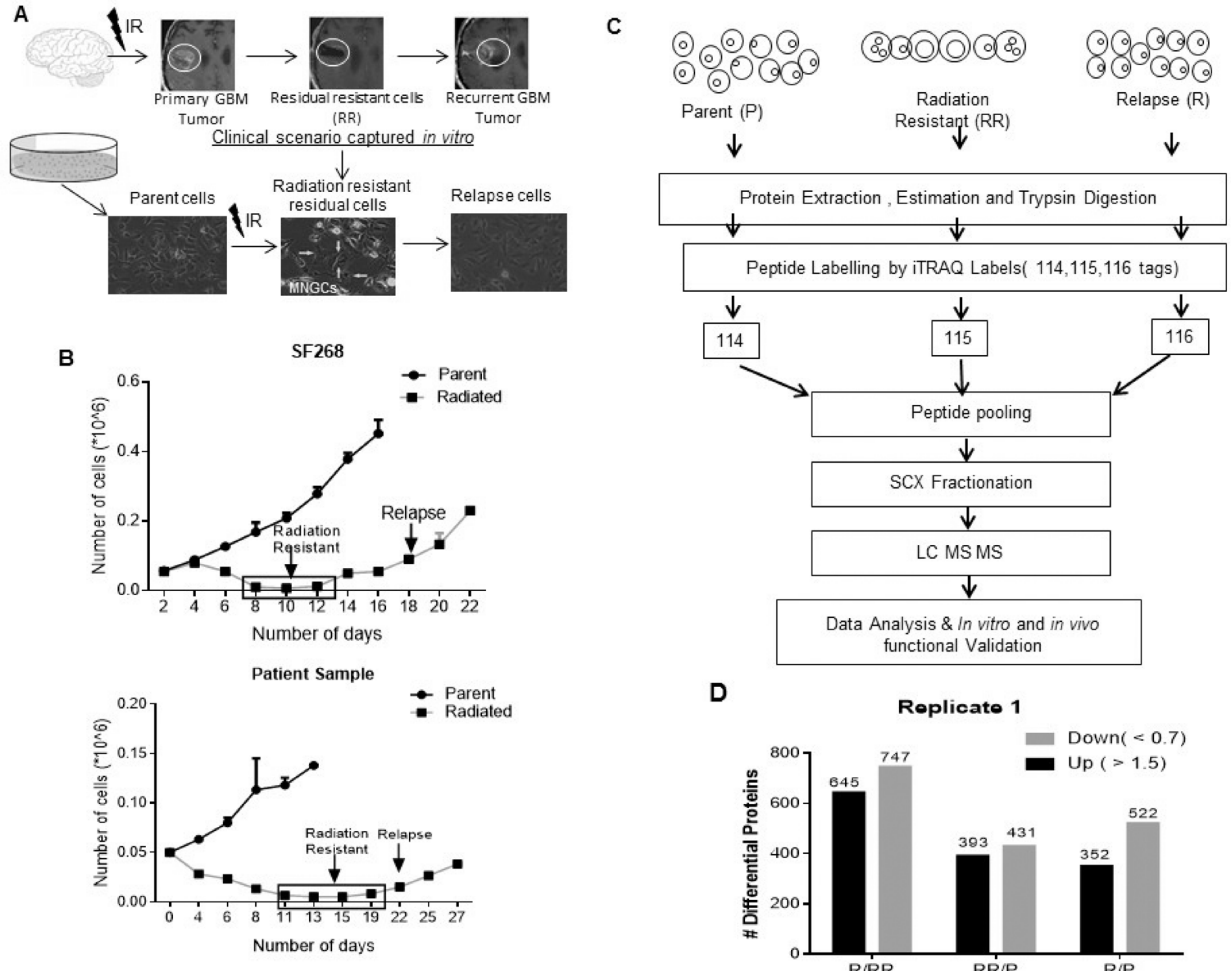
*In vitro* radiation resistant model (**A**) The illustration depicts the clinical scenario in patient’s pre and post treatment in which post-surgery there is a significant regression or complete abolishment of the tumor observed. However, in >90% cases tumor recurs. This clinical scenario was recapitulated in an *in vitro* model. The images represent the SF268 Parent, innate Radiation Resistant (RR) enriched with multinucleated giant cells (MNGCs) and Relapse (R) population. (**B**) Graph represents the growth kinetics of SF268 and Patient Sample post lethal dose of radiation. (**C**) A schematic representation of the proteomics workflow. (**D**) Graphical representation of the number of differential proteins identified in the RR and R w.r.t P and R w.r.t RR by the proteomic analysis. Results in each bar graph are the composite data from three independent experiments performed in triplicate (mean ± SEM)

To capture and understand the survival mechanisms of residual resistant cells of GBM, that are diagnostically undetectable post treatment, we generated *in vitro* radiation resistant model derived from cell lines and patient samples [9] (Figure 1A). Using the same protocol, in this study first the glioblastoma cell lines (SF268 and U87MG) and two short term primary cultures of patient samples (PS1 and PS2) were subjected to their respective lethal dose of radiation (6.5 Gy, 8 Gy, 6 Gy, 6.5 Gy) as determined previously using clonogenic assay [9]. Post treatment initially the cells proliferate, but after 4–5 days post treatment more than 90% cells died leaving behind a small population (<10%) surviving cells. These cells are the innately radiation resistant residual cells (RR) which remain viable but non-proliferative for approximately 7–10 days and acquire Multinucleated Giant (MNGCs) phenotype. However, instead of undergoing mitotic catastrophe, RR cells resume growth to form the relapse (R) population. Figure 1B shows graphs for SF268 and PS1 growth pattern of RR cells. The parent (P), innately radiation resistant (RR) and relapse (R) cells obtained from SF268 were then subjected to quantitative proteomic analysis. The three populations obtained from U87MG, PS1 and PS2 were used for validation and functional studies.

### Quantitative proteomic analysis radio resistant (RR) and relapse (R) cells

**Figure 2:**
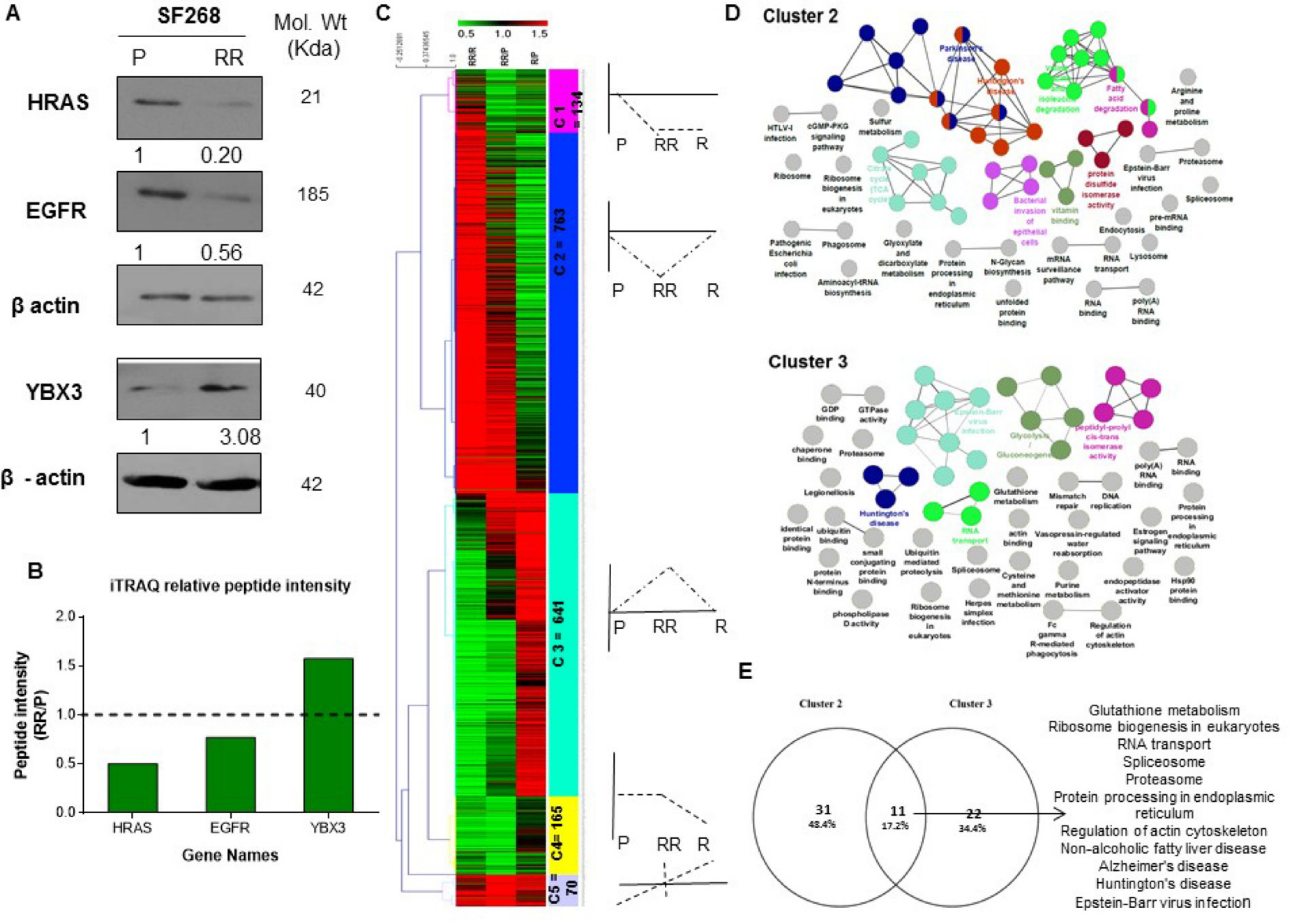
Proteomic analysis of the parent, radiation resistant and relapse population (**A**) Western blots showing the expression of HRas, EGFR, YBX3 in Parent (P), Radiation Resistant (RR) and Relapse (R) population of SF268 cell line. β-actin was used as loading control. (**B**) Bar plot of the relative peptide intensity values of the mentioned proteins in RR/P and R/P as determined by iTRAQ. (**C**) Heat map representation of unsupervised hierarchical clustering of the proteins based on their relative peptide intensities in R w.r.t RR, RR w.r.t P and R w.r.t P. Red- Up-regulation >1.5, Green- Down-regulation <0.5. Heat map is divided into clusters with a dotted plot representing the expression pattern of proteins in each cluster. (**D**) Pathway analysis of the Genes in cluster 2 and cluster were collapsed into pathways using ClueGo and CluePedia plugin of Cytoscape with KEGG and REACTOME pathway databases. Each coloured circle represents a pathway enriched with upregulated and downregulated protein in the RR cells but non-differential in the R cells. (**E**) Venn diagram for the overlap of pathways between cluster 2 and cluster 3

iTRAQ based quantitative proteomic analysis was performed on parent, RR and R cell population of SF268. Figure 1C illustrates the proteomics workflow. Equal amounts of protein from the Parent, RR and R populations was digested with trypsin and their tryptic peptides were labelled with 114, 115 and 116 isobaric reagents respectively for differential protein expression analysis. The iTRAQ-labelled peptide samples were pooled, fractionated and analyzed by LC-MS/MS. The data obtained was searched against National Centre for Biotechnology Information RefSeq database (version 52 40) using Protein Discoverer (version 1.4) using MASCOT and SEQUEST. Compared to parent cells 824 proteins were found to be differentially expressed in RR cells compared to parent cells out of which 393 proteins were up-regulated (fold change >1.5) and 431 proteins were downregulated (fold change <0.7) while 874 proteins were differentially expressed in relapse population of which 352 proteins were up-regulated (>1.5) and 522 proteins were downregulated (<0.7). 1,392 proteins were differentially regulated in R vs. RR out of which 747 proteins were upregulated (>1.5) and 645 were downregulated (<0.7) in the R population (Figure 1D). iTRAQ data was validated by analysing the expression levels of HRAS, EGFR, YBX3 (Figure 2A). Relative peptide intensity values of the three proteins from mass spectrometry showed concurrent expression with the western blot data (Figure 2B).

### Unsupervised clustering of proteomics data identifies protein clusters uniquely differential in each population

Since a cell’s phenotype is an outcome of a collective network of biological processes, it was hypothesized that proteins showing similar expression pattern will participate in similar biological processes. Therefore, we first identified the proteins showing co-expression, for which unique master differential gene list was compiled the at least one of the three binary comparison (RR Vs. P, R Vs. P, R Vs. RR) which comprise of 1773 genes. Unsupervised clustering was performed for these genes based on their respective relative protein abundance values as represented in a heat map. The expression pattern of each cluster is illustrated as a line plot (Figure 2C). Analysis segregated the data set into five clusters (C1-C5) out of which two major clusters, cluster 2 and cluster 3 represented proteins that were exclusively enriched with uniquely downregulated and upregulated proteins in the RR population, respectively. Cluster 2 represents 783 proteins and Cluster 3 represents 641 proteins. Clusters 1, 4 and 5 comprised of proteins that showed similar expression pattern in RR and R cells. 134 proteins were found to be downregulated in the RR and R as compared to the parent cells (cluster 1). The expression of 165 proteins remains at a basal level in the P and RR population however their expression declines in the R cells (cluster 4) and 70 proteins show an escalation in expression in the RR and R as compared to the P cells (cluster 5). Since we were interested to know how the RR cells survive, we focused on the proteins classified in cluster 2 and cluster 3 which comprised of proteins uniquely downregulated and upregulated in the RR cells, respectively.

### Pathway analysis reveals deregulation of proteasome and protein turnover machinery proteins in RR population

**Figure 3:**
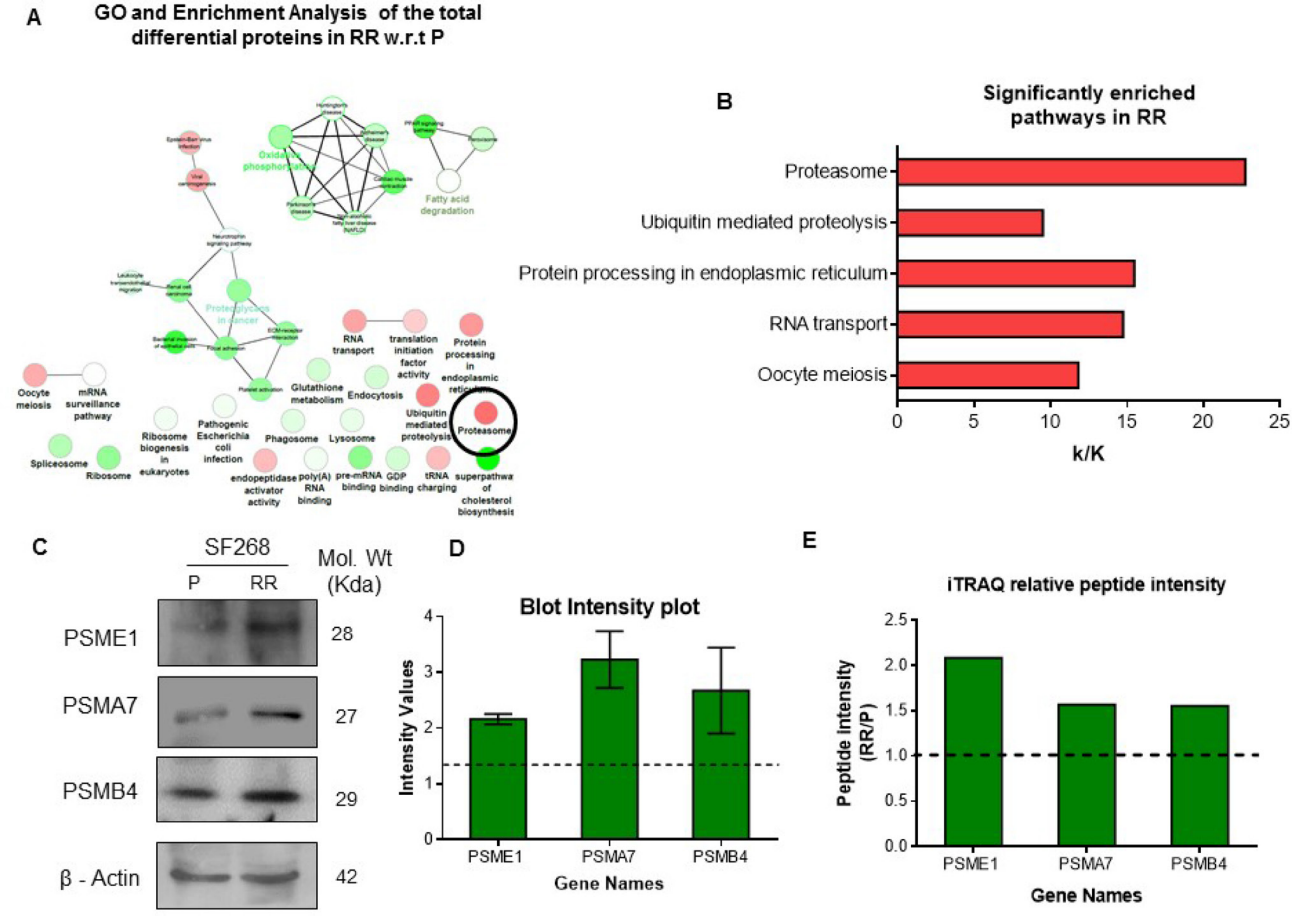
Deregulation of proteasome pathway in the radiation resistant population (**A**) Pathway analysis of deregulated genes in Radiation Resistant (RR) vs. Parent (P) Genes deregulated in RR w.r.t P were collapsed into pathways using ClueGo and CluePedia plugin of Cytoscape with KEGG and REACTOME pathway databases. The colour gradient shows the number of genes of each group associated with the pathway. Equal proportions of the two clusters are represented in white. (**B**) KEGG pathways enriched with upregulated proteins according to their k/K ratio. k–Number of genes identified from the pathway, K–Total number of genes curated in the KEGG database for a pathway. (**C**) Western blot showing the expression of PSME1, PSMA7 and PSMB4 parent (P), Radiation Resistant (RR) and Relapse (R) cells of SF268. β-actin was used as loading control. (**D**) Band intensity plot for the proteins validated by western blot using IMAGE J software. (**E**) Shows the relative peptide intensity values of the three proteins from iTRAQ analysis.

To analyze the molecular pathway that might be involved in the survival and radiation resistance mechanisms of RR cell, pathway enrichment analysis of the deregulated proteins in RR population compared to parent population in cluster 2 and cluster 3 was done using KEGG and REACTOME database (Figure 2D). In total 42 pathways were deregulated in cluster 2, 33 pathways were deregulated in cluster 3. Interestingly, 11 pathways were commonly deregulated in both cluster 2 and 3 (Figure 2E). These pathways included glutathione metabolism, ribosome biogenesis in eukaryotes, RNA transport, spliceosome, and proteasome, protein processing in endoplasmic reticulum, regulation of actin cytoskeleton, non-alcoholic fatty liver disease (NAFLD), Alzheimer’s disease, Huntington’s disease and Epstein - Barr virus infection. Additionally, gene ontology and enrichment analysis of the entire differential proteins found in the RR compared to the parent cells, revealed 24 pathways enriched with upregulated (red circle) and downregulated proteins (green circle). Of these, 8 pathways were enriched with upregulated proteins and 16 pathways were enriched with downregulated proteins (Figure 3A). Out of the 8 pathways that were enriched with upregulated proteins, 5 statistically significant (Term *P* value < 0.05) pathways included Proteasome (8 proteins), Ubiquitin mediated proteolysis (10 proteins), Protein processing in Endoplasmic Reticulum (18 proteins), RNA Transport (17 proteins), oocyte meiosis (9 proteins). However, proteasome pathway was the most deregulated pathway based on the associated genes filter (k/K ratio). Proteomic analysis from three biological replicates also revealed significant deregulation of proteasome pathway in the RR population (Supplementary Figure 2 and Figure 3B). The data sets of all the replicates have been deposited to the ProteomeXchangeConsortium (http://proteomecentral.proteomexchange.org) via the PRIDE partner repository. The internal ID of submission is: px-submission #265394. A ProteomeXchange accession number will be generated after it has been loaded into the database. Proteasome subunits differential in all the four biological replicates have been represented in Table 1. Three subunits PSME1, PSMA7 and PSMB4 were used for validation by western blot (Figure 3C–3E).

**Table 1:** Represents the list of differential proteins identified in the proteasome pathway

REPLICATE 1				
Gene Symbol	Protein Description	Σ^#^ Unique Peptides	Σ^#^ PSMs	Fold Change in RR/P
PSME1	proteasome activator complex subunit 1 isoform 1 [Homo sapiens]	4	4	2.085
PSMD7	26S proteasome non-ATPase regulatory subunit 7 [Homo sapiens]	3	6	1.977
PSMA1	proteasome subunit alpha type-1 isoform 3 [Homo sapiens]	1	2	1.634
PSMD2	26S proteasome non-ATPase regulatory subunit 2 [Homo sapiens]	9	12	1.632
PSMA7	proteasome subunit alpha type-7 [Homo sapiens]	4	13	1.568
PSMB4	proteasome subunit beta type-4 [Homo sapiens]	2	4	1.550
PSMC1	26S protease regulatory subunit 4 [Homo sapiens]	6	10	1.518
PSMA3	proteasome subunit alpha type-3 isoform 2 [Homo sapiens]	2	4	0.656
PSMD14	26S proteasome non-ATPase regulatory subunit 14 [Homo sapiens]	3	4	0.593
REPLICATE 2				
PSMD9	26S proteasome non-ATPase regulatory subunit 9 isoform 1	4	6	1.88
PSMD10	26S proteasome non-ATPase regulatory subunit 10 isoform 1	6	9	1.523
PSMC1	26S protease regulatory subunit 4	19	57	1.381
PSMC6	26S protease regulatory subunit 10B	16	48	1.356
PSMD8	26S proteasome non-ATPase regulatory subunit 8	10	21	1.356
PSMA4	proteasome subunit alpha type-4 isoform 1	10	35	1.294
PSME2	proteasome activator complex subunit 2	12	30	1.281
PSMD13	26S proteasome non-ATPase regulatory subunit 13 isoform 1	19	47	1.243
PSMD7	26S proteasome non-ATPase regulatory subunit 7	10	19	1.227
PSMD12	26S proteasome non-ATPase regulatory subunit 12 isoform 1	22	44	1.207
REPLICATE 3				
PSMD9	26S proteasome non-ATPase regulatory subunit 9 isoform 1	5	7	3.587
PSMC5	26S protease regulatory subunit 8 isoform 1	21	54	1.525
PSMB10	proteasome subunit beta type-10 precursor	1	1	1.445
PSME2	proteasome activator complex subunit 2	9	29	1.41
PSMD6	26S proteasome non-ATPase regulatory subunit 6 isoform 2	19	30	1.382
PSMD4	26S proteasome non-ATPase regulatory subunit 4	12	27	1.362
PSMA3	proteasome subunit alpha type-3 isoform 1	9	25	1.326
PSMD8	26S proteasome non-ATPase regulatory subunit 8	9	19	1.321
PSMC6	26S protease regulatory subunit 10B	18	52	1.318
PSMD13	26S proteasome non-ATPase regulatory subunit 13 isoform 1	17	43	1.302
PSMB7	proteasome subunit beta type-7 precursor	5	17	1.278
PSMD2	26S proteasome non-ATPase regulatory subunit 2 isoform 1	31	74	1.257
PSMD14	26S proteasome non-ATPase regulatory subunit 14	13	23	1.222
PSMC4	26S protease regulatory subunit 6B isoform 1	17	49	1.217
REPLICATE 4				
PSMD9	26S proteasome non-ATPase regulatory subunit 9 isoform 1	6	10	1.95
PSME2	proteasome activator complex subunit 2	9	35	1.77
PSMD8	26S proteasome non-ATPase regulatory subunit 8	11	22	1.579
PSMD4	26S proteasome non-ATPase regulatory subunit 4	12	26	1.489
PSMD7	26S proteasome non-ATPase regulatory subunit 7	11	23	1.411
PSMC4	26S protease regulatory subunit 6B isoform 1	23	70	1.382

Columns from the right represent the gene symbol, protein description, ^#^- number of unique peptides identified, number of peptide score matches (PSMs) and the fold change of the proteins in RR w.r.t P.

### RR cells display enhanced proteasome activity and survival dependency on proteasome activity *in vitro*

**Figure 4:**
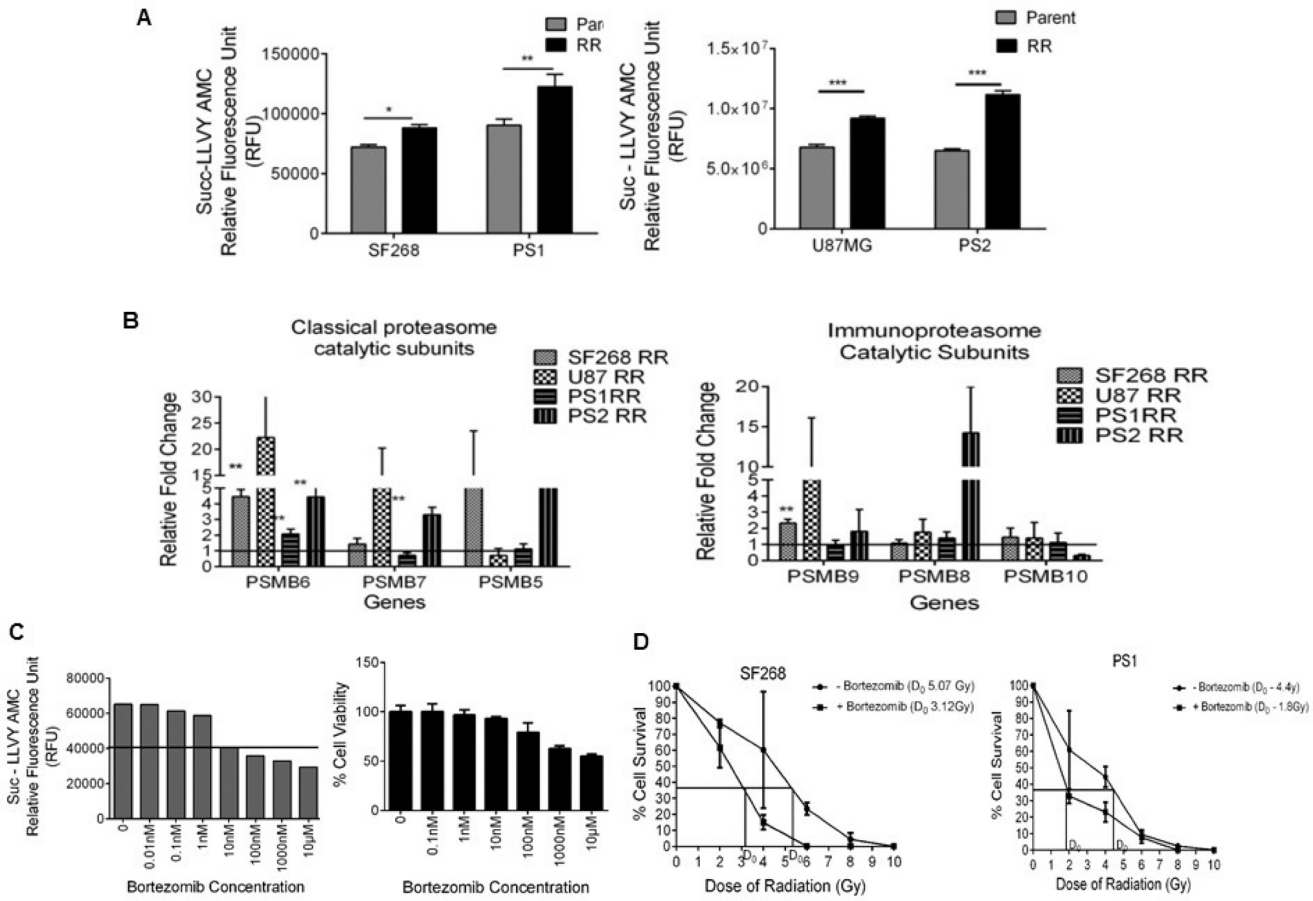
RR cells display enhanced proteasome activity and survival dependency on proteasomes *in vitro* (**A**) Data represents the chymotrypsin like proteasome activity measured using Succ-LLVY AMC florigenic substrate in the P and RR population of SF268, U87MG, PS1 and PS2. (**B**) The graph depicts the RPL19 normalised mRNA levels of classical and Immunoproteasome proteasome beta catalytic subunits respectively in the RR population of SF268, U87MG, PS1, and PS2 compared to the parent population. (**C**) Proteasome activity inhibition and % cell viability at different concentrations of proteasome inhibitor–Bortezomib in SF268. (**D**) Graph shows percentage of cells of SF268 and PS1 surviving at different doses of γ radiation with and without 10 nM Bortezomib in a clonogenic assay. (D) Bar graph represents the percentage of viable cells (at 72 hrs) as assessed by MTT assay at different concentrations of Bortezomib. Cells were treated with Bortezomib for 12 hrs. Results in each bar graph are the composite data from three independent experiments performed in triplicate (mean ± SEM); ^***^P = 0.001c)

Since the RR population exhibited increased protein expression of proteasome subunits, we sought to observe if the expression correlated with proteasome activity. Therefore, proteasome activity was analysed in the parent and RR cells of SF268, U87MG, PS1 and PS2 using florigenic substrate Suc-LLVY-Amc. Indeed the RR population of SF268, U87MG, PS1 and PS2 showed 22.18%, 35.60%, 20.63% and 71.63 % increase respectively in the proteasome activity compared to the parent cells (Figure 4A). Among the 9 subunits overexpressed in the RR, 3 subunits are part of the 19S regulatory subunit–PSMC1, PSMD2, PSMD7;3 subunits of the 20 S core particle–PSMA1, PSMA7, PSMB4 and 1 subunits of the 11 S regulatory subunits–PSME1. Most of the subunits belong to the classical proteasome. Hence the transcript levels of beta catalytic subunits: PSMB6 (β1- caspase like activity), PSMB7 (β2-trypsin-like activity) and PSMB5 (β5-chymotrypsin-like activity), were checked. PSMB6 transcript levels were elevated in the RR population of all the samples, PSMB7 and PSMB5 were elevated in at least one cell line and one patient sample. Proteomics data also identified a regulatory subunit of immunoproteasome (PSME1). Therefore, the mRNA levels of its catalytic subunits PSMB9, PSMB8 and PSMB10 were also determined (Figure 4B). However, the transcript levels of the three subunits were not significantly high in any of the samples.

**Figure 5:**
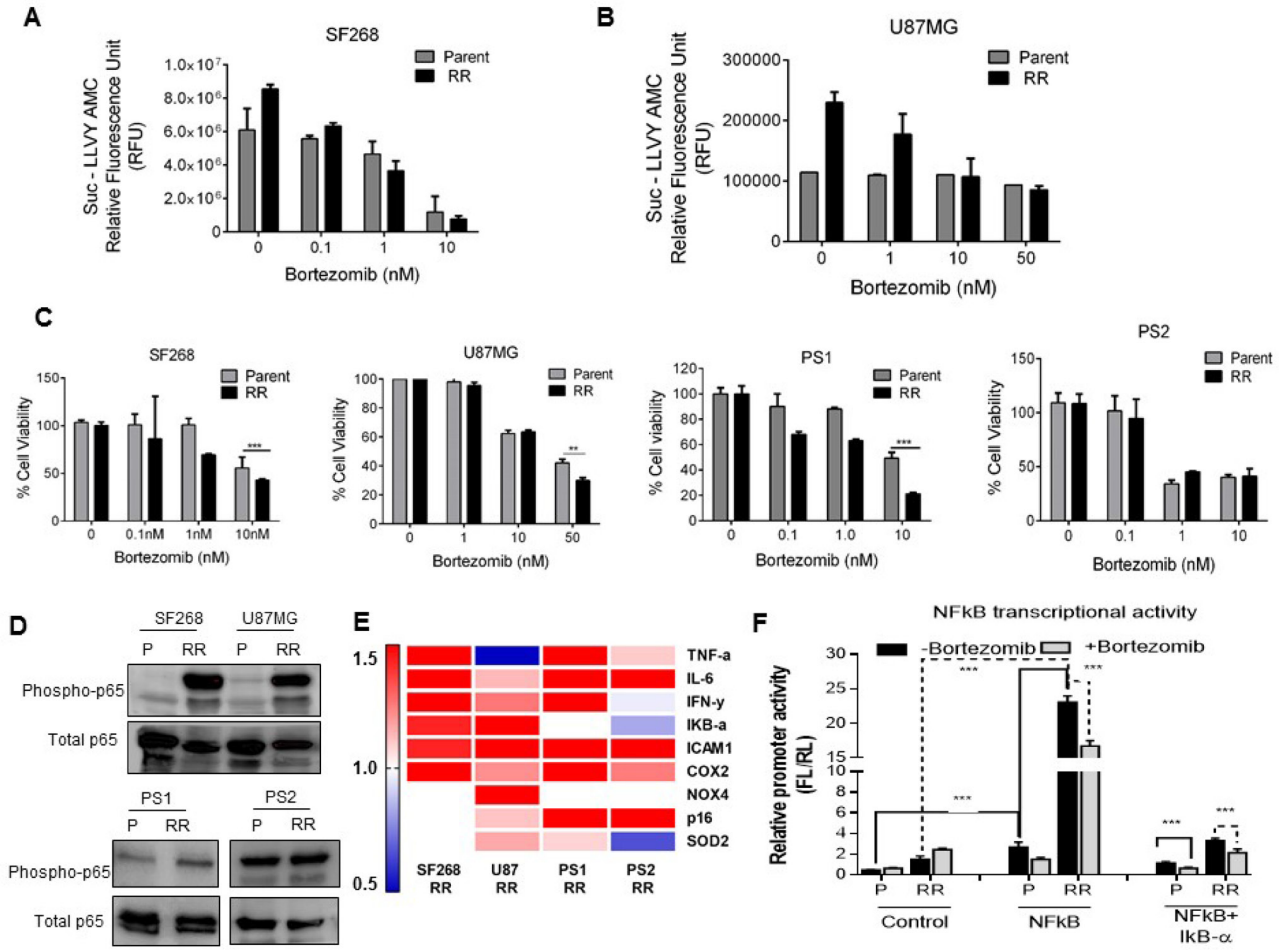
Proteasomes indirectly regulate RR cell survival via the NFkB activation (**A** and **B**) Bar graph shows proteasome activity in parent and RR cells of SF268 and U87 at different concentrations of the Bortezomib as mentioned. (**C**) Bar graph represents the percentage of viable cells (at 72 hrs) as assessed by MTT assay at different concentrations of Bortezomib. Cells were treated with Bortezomib for 12 hrs. Results in each bar graph are the composite data from three independent experiments performed in triplicate ((mean ± SEM); ^***^P = 0.001) (**D**) Western blot represents the expression of phosphor- p65 in the P (Parent) and RR (Radiation resistant) cells of SF268, and U87MG, PS1 and PS2. Total (T) total- p65 levels were used as loading controls. (**E**) Heat map representation of gene expression values NFkB target genes by qPCR in the RR population of SF268, U87, PS1 and PS2 compared to the parent population. GAPDH was used as internal control. Results are the composite data from three independent experiments performed in triplicate (mean ± SEM); ^*^P = 0.05, ^**^P = 0.01 and ^***^P = 0.001 (**F**) Bortezomib treatment repressed the transcriptional activity of NFkB promoter luciferase constructs. The NFkB firefly luciferase construct was transfected into Parent and RR cells and then treated with Bortezomib as indicated. As a control Con A control plasmid was transfected with Renilla luciferase construct. The pTRIPZ IkB-alpha construct was used as NFkB suppressor. Luciferase values subsequent to normalization were plotted.

Since the RR population exhibited increased proteasome activity we wanted to analyze if the survival of RR cells was dependent on the proteasome activity. For this we used bortezomib (BTZ), a pharmacological inhibitor of proteasome routinely used in the treatment of multiple myeloma. First we determined the concentration of bortezomib at which proteasome activity was maximally inhibited with minimal cellular toxicity. For this proteasome activity of SF268 was assessed after 12 h. treatment of bortezomib at different concentrations (0.01 nM to 1000 nM). As seen from Figure 4C, 10 nM of bortezomib was the minimum concentration at which significant inhibition of proteasome activity was observed and there was no significant cell death in RR as compared to parent. Once the non-toxic concentration of bortezomib on parent cells was determined, we wanted to see if the inhibition of proteasome sensitizes the glioma cells to radiation. SF268 and PS1 cells were treated for 12 hrs with 10 nM bortezomib and their % cell survival was recorded at different doses of radiation. As shown in Figure 4D, bortezomib treatment significantly reduced the D_0_ dose of radiation from 5.07 Gy to 3.12 Gy and 4.4 Gy to 1.08 Gy for SF268 and PS1 respectively, showing that proteasome inhibition radio sensitizes glioma cells. We then wanted to analyse the effect of bortezomib on RR population that have higher proteasome activity. For this the parent and RR population of SF268 and U87 were treated with 0.1 nM, 1 nM and 10 nM concentrations of bortezomib for 12 hrs. Following the treatment cells were monitored for proteasome activity. Both, parent and RR cells showed a gradual decrease in the activity of proteasomes with increasing concentration of the drug (Figure 5A and 5B). However, 72 hours post drug treatment RR cells were significantly (8% SF268, 10% U87 and 23% PS1) more sensitive to proteasome inhibition compared to the parent population. PS2 showed similar % reduction in viability as compared to the parent population at 10 nM (Figure 5C).

### Proteasomes indirectly regulate RR cell survival via the NFkB activation

We further wanted to determine if the proteasome targets were down-regulated in the RR population due to degradation via ubiquitin mediated proteasome pathway. Down regulated proteins were analysed for presence of annotated ubiquitin binding lysine residues. These proteins were downloaded from Uniprot database [29] and parsed using in-house python scripts to determine presence of curated ubiquitin binding sites. Of the 431 proteins, 14 proteins were found to harbour lysine residues which can undergo ubiquitin modification (Supplementary Figure 1). One of the well-known substrates of the 26 S proteasome is IκB-α which upon degradation leads to the activation of the transcription factor NFkB. An increased proteasome activity should modulate the levels of activated NFkB in the RR population. Therefore, we checked for the levels of activated NFkB by western blot in the P and RR cells of cell lines and patient samples. Indeed, the RR cells displayed increased levels of activated NFkB in both the cell lines and PS1 (Figure 5D). Furthermore, the transcript levels of 9 NFkB target genes (TNF-α, IL6, IkB-a, IFN-γ, ICAM1, COX2, NOD4, p16, SOD2) were screened in RR cells of the cell lines and patient sample by real-time PCR. A heat map representation of the 9 genes depicts upregulation of at least 6 genes out of the 9 in SF268, U87 and PS1 which also harbour increased expression of phospho-NFkB suggesting the presence of a transcriptionally active NFkB in RR cells (Figure 5E). To directly assess the NFkB transcriptional activity in the RR cells of U87, we monitored the relative promoter activity of the luciferase based NFkB reporter constructs in the P and RR cells. The RR cells showed a significant increase (20 fold) in NFkB transcriptional activity as compared to the parent population (P). Importantly, administration of the proteasome inhibitor (Bortezomib) in the P and RR cells diminished this activity by 1.5 and 3.0 fold demonstrating the dependency of NFkB activity on the proteasome activity. A synergistic inhibitory effect was observed in the presence of IkB-alpha construct and bortezomib in the P and RR cells. However, the RR cells displayed a much higher reduction as compared to the P cells (Figure 5F).

### Inhibition of proteasome activity inhibits tumour formation and *in vivo*

**Figure 6:**
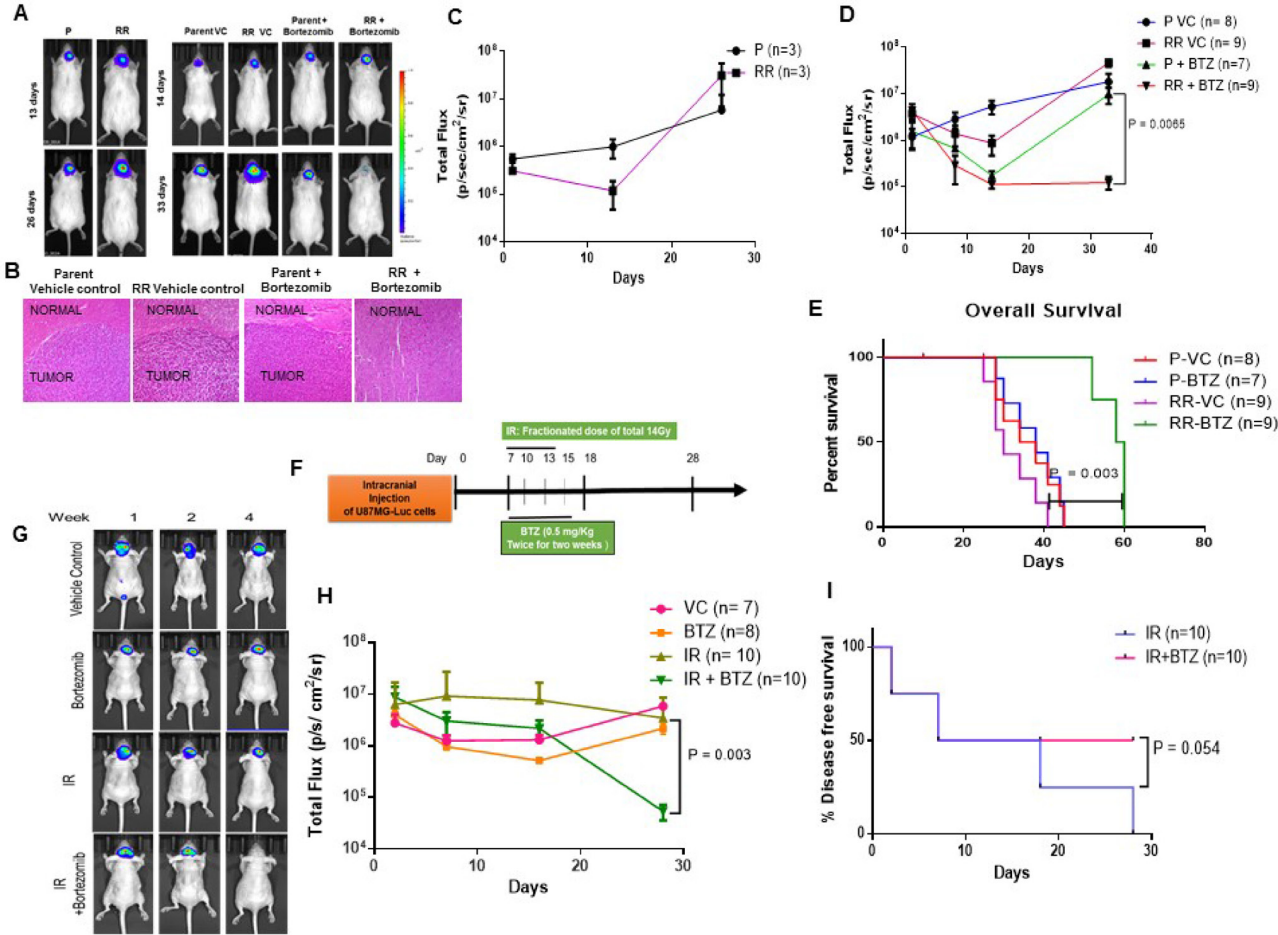
Proteasome inhibition reduces the tumorigenic potential of the cells *in vivo* (**A**) Left panel - Representative bioluminescence images after orthotopic injection of U87MG-Luciferase labelled Parent (P) and Radiation Resistant (RR) cells. Right Panel - Bioluminescent images after orthotopic injection of U87MG-Luciferase labelled Parent (P) and Radiation Resistant (RR) cells treated with Vehicle Control (VC) and Bortezomib. (**B**) Hematoxylin and eosin (H&E) staining of mice brain slices. Brain slices of the brain tissue from mice injected with Parent Vehicle control, RR Vehicle Control, Parent + Bortezomib, RR + Bortezomib cells were formalin fixed and paraffin embedded. Sections stained with H&E show regions infiltrated with tumour cells. All photomicrographs are shown with the same magnification. Bar = 100 μm. (**C**) Graph represents bioluminescence signal at different days post injection in mice injected with P and RR cells. (**D**) Graph represents bioluminescence intensity at different days post injection of mice injected with P and RR cells pretreated with bortezomib as compared to P and RR cells treated with vehicle control. ‘n’ represents number of mice per group. (**E**) Kaplein Meier Curve for the overall survival of the mice in the pretreated study. (**F**) Schematic representation for studying the effect of intraperitoneal injections of bortezomib along with radiation treatment of mice intracranially injected with parent GBM cells. IR–Radiation; BTZ–Bortezomib. (**G**) Representative bioluminescence images of tumor formation in the mice treated with IR and BTZ compared to the mice which were administered with Vehicle Control (VC), only BTZ and only IR. (**H**) Graphical representation of bioluminescence intensity recorded for mice treated with IR and BTZ compared to the mice which were administered only saline as Vehicle Control (VC), only BTZ, only IR. (**I**) Kaplein Meier Curve for % tumor free animals in the radiation and intraperitoneally administered BTZ study.

We have shown that radiation resistant residual (RR) cells formed in our *in vitro* radiation resistant model systems retain their tumorigenic potential and re-grow to give rise to recurrent tumour. We first wanted to analyze if the RR cells are capable of forming tumour *in vivo* as well. For this pLenti6-luc2 U87MG cells [30] stably expressing luciferase were treated with the lethal dose of radiation 8Gy and RR cells were collected. The parent and RR cells were then stereo tactically injected in the brain of 6–8 weeks old NOD/SCID mice. Tumour growth was monitored using bioluminescence imaging. As seen from Figure 6A left panel and Figure 6C, RR cells were able to give rise to tumours and had greater tumorigenic potential as compared to the parent cells.

We then evaluated the effect of proteasome inhibition on the tumorigenicity of the parent and RR cells. Since U87MG cells showed higher proteasome activity than the SF268 (Figure 4A), hence they also required a higher concentration of bortezomib (50 nM) for reducing the viability of their RR. Therefore for *in vivo* studies U87MG parent and RR cells were treated with 50 nM bortezomib for 12 hrs prior to injection. Tumour formation was monitored by bioluminescence. As expected at day 14 post injection parent and RR cells treated with vehicle control or bortezomib showed almost similar growth, however, by day 33 while the parent cells treated with bortezomib had formed large tumours, the RR cells treated with bortezomib showed significant reduced bioluminescence intensity (Figure 6A, right panel). Presence of tumour cells was seen with Haematoxylin and Eosin staining in the brain slices of all the treatment groups of mice except for the brain tissue of mice treated injected with RR cells + bortezomib (Figure 6B). As represented in Figure 6D, the mice injected with bortezomib treated RR cells showed a significant decline in bioluminescence as compared to the group injected with bortezomib treated P cells. Also, the overall survival of this group (RR-BTZ) was significantly higher than that of the other three groups as shown in Figure 6E. Median survival of each group are as follows: P- VC–36 days, P–BTZ–38 days, RR–VC–30 days, RR–BTZ–58 days. Further, we did intracranial injection of parental cells followed by radio therapy (fractionated dose of 14 Gy) followed by intraperitoneal injection of bortezomib (0.5 mg/Kg twice in a week for two weeks) as depicted in Figure 6F. Representative bioluminescence images from each group are shown in Figure 6G. The results show a significant reduction in bioluminescence of animals treated with radiation along with BTZ as compared to the radiation alone group (Figure 6H). The disease free survival of mice was significantly higher in the group treated with radiation and BTZ as compared to radiated alone group (Figure 6I).

Together these data confirmed that the proteasome inhibition *in vitro* and *in vivo* resulted in tumour reduction and abrogation of relapse.

## DISCUSSION

Radio resistance and recurrence is currently an inevitable consequence in the field of glioblastoma. Until now, the mechanisms of radio resistance in glioblastoma have been explored in *in vitro* and in *in vivo* settings either immediately post radiation or after generation of repeated doses of radiation (acquired resistance) but not in the residual radiation resistant cells. However, in this study we focused on the processes deregulated in the innately radiation resistant residual (RR) population as we have previously shown that these are the cells responsible for relapse in glioblastoma [9]. We performed iTRAQ based quantitative proteomic analysis on the parent (P), innately radiation resistant residual (RR) and relapse (R) population. Amongst the many pathways, we found the proteasome pathway to be most significantly deregulated in the RR cells.

Proteasomes are well known targets in cancer therapy owing to their role in maintaining homeostasis of proteins involved in cell cycle, signalling pathways regulating cell survival and apoptosis [31–34]. Cancer cells harbour enhanced proteasome activity compared to their normal counterparts but the exact reason for this surge is still unknown. It is speculated that this escalation in proteasome activity is to cope with crisis such as mutational events and chromosomal instabilities. Although proteasomes are identified as direct targets of radiation, their inhibition is short lived and thus the need for drugs targeting their enzymatic activity [28, 35, 36]. Lower proteasome activity is shown to be a marker for tumour initiating cells and stem cells [37]. Proteasomes are also found to be downregulated in radio-resistant cells of breast cancer and prostate cancer established *in vitro* [27, 35, 38]. Contrary to these reports, we observed an enhanced expression and activity of proteasomes in the innate radio-resistant residual cells of glioblastoma. Subsequently, we also identified 14 out of 431 downregulated proteins that harbour ubiquitin binding lysine residues (Supplementary Figure 1). These proteins in the RR cells, we predict to be either ubiquitin adapters or direct targets of the ubiquitin mediated proteasome machinery. This reduced number of proteins with ubiquitin binding attributes to the fact that proteasomes degrade a significant cellular portion by a ubiquitin independent manner also which is still incompletely understood [39].

**Figure 7:**
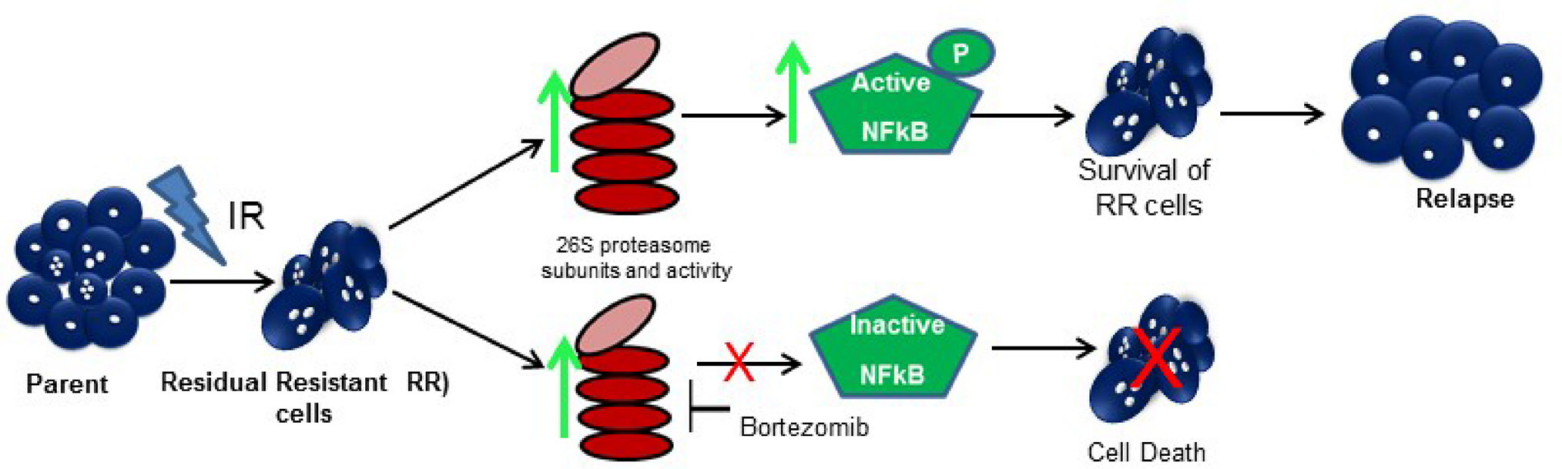
Proposed model for the study

Bortezomib preferentially inhibits the chymotrypsin like activity of proteasomes and is currently being used in the treatment for multiple myeloma [28, 40, 41]. In GBM, it has been reported to sensitize the parent GBM cells to temozolomide and radiation treatment but after immediate exposure to the drug and radiation [42]. However, in our study we show that the residual resistant cells that are formed after a period of 5–7 days post radiation are more sensitive to proteasome inhibition compared to the parent cells, although, there is a differential response to proteasome inhibition amongst the cell lines (SF268, U87MG) and patient samples (PS1 & PS2) as depicted in Figure 5C. This could be due to the heterogeneity of GBM tumours. The subtle effect of bortezomib seen *in vitro* after 72 hrs post treatment is significantly enhanced in reducing tumorigenicity of the treated cells *in vivo,* suggesting a slow and prolonged effect of proteasome inhibition on the survival of the cells. A significant effect of proteasome inhibition was observed on the overall survival of mice which were injected with pre-treated RR-BTZ cells along with an increased % of tumour free mice when BTZ was administered intraperitoneally along with radiation as shown in Figure 6H and 6I. The increased levels of activated NFkB and its transcriptional activity in the RR cells correlate with previous reports where NFkB has been shown to promote radio resistance in Glioblastoma and other cancers. It has been reported to trigger pro-survival and anti-apoptotic signals by transcriptional activation of over 200 genes including the pro inflammatory cytokines, cell-cell adhesion molecules. We have observed cytokines such as TNF-α, IFN-γ, IL-6 and antioxidant genes such COX2 levels increased in the RR. Its activation can occur via IkB-α degradation (Classical pathway) or the by TNF-α (alternative pathway) [43–45]. However, the exact mechanism downstream to higher proteasome expression and NFkB activity in the RR cells needs to be further explored. Nonetheless, this study as illustrated in Figure 7, establishes that proteasomes aid the survival of the innate radiation resistant population via NFkB pathway and hence can be valuable targets for precluding relapse in glioblastoma.

## MATERIALS AND METHODS

### Cell culture and drug treatment

GBM grade IV cell lines U87MG and SF268 were obtained from ATCC in 2011. These cell lines were last authenticated in the laboratory by short tandem repeat profiling based on eight markers in May 2014. The cell line was maintained in DMEM containing 10% (v/v) FBS, penicillin (200 U/ml), streptomycin (100 μg/ml) and incubated at 37° C in a humidified incubator with an atmosphere of 50 mL/L CO_2_. Proteasome inhibitor was obtained from NATCO.

### Cell synchronization and radiation treatment

The cells growing in 10% FBS containing media were washed with 1X PBS. The cells were incubated with 0.05% FBS containing DMEM for 72 hrs. After 72 hrs, cells were replaced by 10% FBS containing median and were irradiated using 60 Co γ-rays at the respective lethal dose.

### Protein extraction

10 million cells of the Parent (P), Radiation Resistant (RR) and Relapse (R) cells were grown under normal growth conditions. The media was aspirated and the cells were washed thrice with cold 1 X PBS after which the cells were scraped and pelleted down. The cell pellet was suspended in 150 µl of 0.5% SDS Solution and sonicated with 10 pulses each for 10secs. The sonicated cells were centrifuged at 4000 RPM for 15 mins at 4° C and the supernatant was used for the proteomic analysis. The protein concentration was determined using bichoninic acid assay and equal amounts of protein from the 3 conditions were taken for further analysis.

### iTRAQ labelling

Protein extracts from the untreated, radiation resistant and relapse cells were digested with trypsin and the peptides were labelled with iTRAQ reagents according to the manufacturer’s instructions (iTRAQ Reagents Multiplex kit; Applied Biosystems/MDS Sciex, Foster City, CA). Briefly, 80 µg of protein from each sample was reduced, alkylated and digested with sequencing grade trypsin; (Promega, Madison, WI, USA). Peptides from P, RR and R were labelled with iTRAQ reagents containing 114, 115 and 116 reporter ions, respectively. The three labelled samples were pooled, vacuum-dried and subjected to fractionation by strong cation exchange (SCX) chromatography.

### SCX fractionation

The pooled sample after iTRAQ labelling was resuspended in 1 ml of buffer A [10 mM KH2PO4, 25% (v/v) acetonitrile (ACN), pH 2.9] and separated on a SCX column (Zorbax 300-SCX, 5 µm, 2.1 mm ID × 50 mm, Agilent Technologies, Santa Clara, CA, USA) at a flow rate of 700 µl/min with a 40 min gradient [5 min, 0–5% buffer B (buffer A + 350 mM KCl); 5 min, 5–10%; 5 min, 10–23%; 5 min, 23–50%; 10 min, 50–100%; 10 min, 100% B]. One minute fractions were collected, vacuum-dried and desalted using C18 cartridge (Pierce, Rockford, USA) as per the manufacturer’s instructions. After desalting, consecutive fractions were pooled to obtain a total of thirteen fractions for LC-MS/MS analysis.

### LC-MS/MS analysis

Nanoflow electrospray ionization tandem mass spectrometric analysis of peptide samples was carried out using LTQ-Orbitrap Velos (Thermo Scientific, Bremen, Germany) interfaced with Agilent’s 1200 Series nanoflow LC system. The chromatographic capillary columns used were packed with Magic C18 AQ (particle size 5 μm, pore size 100Å; Michrom Bioresources, Auburn, CA, USA) reversed phase material in 100% ACN at a pressure of 1000 psi. The peptide sample from each SCX fraction was enriched using a trap column (75 μm × 2 cm) at a flow rate of 3 μl/min and separated on an analytical column (75 μm × 10 cm) at a flow rate of 350 nl/min. The peptides were eluted using a linear gradient of 7–30% ACN over 65 min. Mass spectrometric analysis was carried out in a data dependent manner with full scans acquired using the Orbitrap mass analyser at a mass resolution of 60,000 at 400 m/z. For each MS cycle, twenty most intense precursor ions from a survey scan were selected for MS/MS and fragmentation detected at a mass resolution of 15,000 at m/z 400. The fragmentation was carried out using higher-energy collision dissociation (HCD) as the activation method with 40% normalized collision energy. The ions selected for fragmentation were excluded for 30 sec. The automatic gain control for full FT MS was set to 1 million ions and for FT MS/MS was set to 0.1 million ions with a maximum time of accumulation of 500 ms, respectively. For accurate mass measurements, the lock mass option was enabled.

### Protein identification and quantitation

The MS data was analyzed using Proteome Discoverer (Thermo Fisher Scientific, Version 1.4). The workflow consisted of a spectrum selector and a reporter ion quantifier. MS/MS search was carried out using SEQUEST and MASCOT search algorithms against the NCBI RefSeq database (release 52 40) containing 31,811 proteins. Search parameters included trypsin as the enzyme with 1 missed cleavage allowed; oxidation of methionine was set as a dynamic modification while alkylation at cysteine and iTRAQ modification at N-terminus of the peptide and lysine were set as static modifications. Precursor and fragment mass tolerance were set to 20 ppm and 0.1.Da, respectively. False Discovery Rate (FDR) was calculated by searching the proteomic data against a decoy protein database. Only those Peptide Spectrum Matches (PSMs) that qualified a 1% FDR threshold were considered for further analysis. Unique peptide (s) for each protein identified was used to determine relative protein quantitation based on the relative intensities of reporter ions released during MS/MS fragmentation of peptides.

### Bioinformatics analysis

Heat Map representation for the differential genes on the basis of their relative peptide intensities was constructed using MeV software (v 4.9.0). Unsupervised Hierarchical clustering of the genes was done using Pearson Correlation method. Functional annotation and Gene enrichment pathway analysis was done using Cytoscape (v 3.5.1) ClueGo (v 1.8) and CluPedia (v 1.0) plugin with default parameters. KEGG and REACTOME pathway databases were used for reference.

### Western blot analysis

Cells were lysed using EBC lysis buffer (120 mM NaCl, 50 mM Tris-Cl (pH 8.0), 0.5% (v/v) Nonidet P-40, 50 μg/ml PMSF and protease, phosphatase inhibitor cocktail for 45 minutes on ice. The supernatant were collected and 40 ug of protein was used for immunoblotting using anti-YBX3 (rabbit; 1:1000; Pierce), anti-PSMB4 (rabbit; 1:1000; Pierce), and anti-PSMD10 (rabbit; 1:1000; Pierce), Actin (Sigma; 1:4000 dilutions), was used as a loading control. Immune-reactive proteins were visualized using an enhanced chemiluminescence (ECL) reagent (Pierce).

### MTT cytotoxicity assay

5000 cells/well were seeded in 96 well plates for overnight. Bortezomib (Bortenat 2 mg; Natco Company) was added at different concentration i.e. 0.1 nM, 1 nM, 10 nM and 100 nM. After 72 hrs 10 μL of MTT reagent (5 mg/ml in PBS, Himedia TC191-1G) was added to each well and incubated for 4 h. Crystals were dissolved using freshly prepared acidified isopropanol containing 10% tritonX-100. Optical density was measured at 570 nM by (SPECTROstar^NANO^star spectrophotometer).

### Proteasome activity assay

0.1 million cells were pelleted, washed twice with 1X PBS and resuspended in ATP buffer containing 50 mM Tris (pH 7.5), 5 mM MgCl2, 1 mMATP, 10% glycerol and protease inhibitor cocktail (Sigma). Cell suspensions were ultra-sonicated for four cycles of 5 s each (with 1 s break after each 2 s) at 30 kHz on ice. Proteasome activity was measured using 50 µM Suc-LLVY-7-amino-4-methyl coumarin substrate and fluorescence readings were taken at excitation 355 nm/emission 460 nm.

### Trypan blue exclusion assay

0.1 million cells from all cultures were seeded in a 24 well plate and irradiated with the lethal dose of radiation. Viable cells from each well were counted every alternative day till 22 days to monitor the cell survival post radiation on a haemocytometer.

### Orthotopic xenograft mouse experiments

All animal experiments were licensed through the Laboratory Animal Facility of ACTREC, TMC. Protocols were reviewed by the Institutional Animal Ethics Committee (IAEC). NUDE/SCID mice (6–8 weeks old) bred and maintained in an isolated facility within a specific pathogen-free environment were used for this study. 1 × 10^5^ pLenti6-luc2 U87MG cells stably expressing luciferase were intracranially injected for generating the orthotopic GBM model and for studying the tumorigenicity of pre-treated Parent and RR cells. 2.5 × 10^5^ pLenti6-luc2 U87MG stably expressing luciferase were intracranially injected for studying the effect of proteasome inhibitor along with radiation. In order to perform intracranial injection, the cells were suspended in 5 µl 1X PBS prior to injection and kept on ice until injected. Prior to injecting the cells intracranially, the mice were anesthetized using an injection mix of Ketamine (120 mg/kg)/Xylazine(mg/kg)/Saline and the mice was placed on the stereotaxic for stereotactic surgery. A 10 mm to 15 mm long incision was made on top of the skull. A small hole was drilled using a sterile 26 gauge sharp needle at 1 mm posterior to bregma and 2 mm lateral to coronal suture and 2.5 mm depth. The 5 µl cell suspension was then loaded onto the Hamilton syringe and injected at a rate of 1 μl per minute for a total of 6–8 minutes. The tumours were allowed to grow and animals were sacrificed using CO_2_ at the onset of disease symptoms, such as weight and activity loss, and the brains were removed.

### Radiation and drug treatment of orthotopic GBM mouse model.

The mice were divided into four groups post 7–10 days of intracranial injection: Vehicle control, bortezomib (Bortenat 2 mg, NATCO company), Radiated group, Radiation and BTZ group. Radiation was delivered to the whole brain of anesthetized mice, immobilized in a plastic chamber using 60Co γ-rays. A total dose of 14 Gy was administered over a period of 7 days. 0.5 mg/Kg of bortezomib was administered intraperitoneally twice in a week for 2 weeks.

### Bioluminescence imaging of orthotopic tumor xenografts

Mice were anaesthetized with Ketamine/Xylazine and were administered luciferin (D-Luciferin potassium salt, 150 mg/kg, Calliper Life Sciences) via intraperitoneal injection. The images were acquired 10–12 minutes post injection. The time chosen was based on the pharmacokinetics of luciferin which defines that maximum luminescence emission and greatest sensitivity of detection will be obtained when cell luminescence is detected after 10–15 mins of injection of luciferin. The selected imaging time was maintained as constant among all the animals to be imaged. Regions of interest encompassing the intracranial area of signal were defined using Living Image software, and the total photons/s/sr/cm^2^ (photons per second per steradian per square cm) was recorded.

### Statistical methods

All data are represented as means ± standard error means (SEMs). The two-tailed Student’s *t*-test was applied for statistical analysis. The Kaplan–Meier curve was plotted to generate the survival curves and to estimate the median survival values. Differences between survival curves were compared using a log-rank test.

## SUPPLEMENTARY MATERIALS FIGURES


