# Correction: Enhanced proteasomal activity is essential for long term survival and recurrence of innately radiation resistant residual glioblastoma cells

**DOI:** 10.18632/oncotarget.28778

**Published:** 2025-11-06

**Authors:** Jacinth Rajendra, Keshava K. Datta, Sheikh Burhan Ud Din Farooqee, Rahul Thorat, Kiran Kumar, Nilesh Gardi, Ekjot Kaur, Jyothi Nair, Sameer Salunkhe, Ketaki Patkar, Sanket Desai, Jayant Sastri Goda, Aliasgar Moiyadi, Amit Dutt, Prasanna Venkatraman, Harsha Gowda, Shilpee Dutt

**Affiliations:** ^1^Shilpee Dutt Laboratory, Tata Memorial Centre, Advanced Centre for Treatment, Research and Education in Cancer (ACTREC), Kharghar, Navi Mumbai, India; ^2^Institute of Bioinformatics, International Technology Park, Bangalore, India; ^3^Advanced Centre for Treatment, Research and Education in Cancer (ACTREC), Tata Memorial Centre (TMC), Kharghar, Navi Mumbai, India; ^4^Integrated Genomics Laboratory, Advanced Centre for Treatment, Research and Education in Cancer, Tata Memorial Centre, Navi Mumbai, Maharashtra, India; ^5^Laboratory Animal Facility, Advanced Centre for Treatment, Research and Education in Cancer (ACTREC), Tata Memorial Centre (TMC), Kharghar, Navi Mumbai, India; ^6^Department of neurosurgery Tata Memorial Centre, Advanced Centre for Treatment, Research and Education in Cancer, Navi Mumbai, India; ^7^Homi Bhabha National Institute, Training School Complex, Anushakti Nagar, Mumbai, India; ^8^Department of Radiation Oncology, Tata Memorial Centre, Advanced Centre for Treatment, Research and Education in Cancer, Navi Mumbai, India

**This article has been corrected:** This article has been corrected: In Figure 6A, the representative bioluminescence image of mouse after 14 days of treatment with Vehicle Control (VC) of the orthotopic Radiation Resistant (RR) tumor is an accidental duplicate of the mouse image with a tumor from parental cells treated for 14 days with Bortezomib. The authors stated that the discrepancy in the representative images must have happened inadvertently during the compilation of Figure 6A and mentioned that because these are representative images this has no bearing on the results or the conclusion of this manuscript. The authors provided raw data which included the original images of the mice for the RR VC (*n* = 8) and Parent+Bortezomib (*n* = 7) groups and Excel sheets with ROI of the tumors. The corrected Figure 6A, which uses the original data and features replaced representative mouse images for both RR VC and Parent+Bortezomib groups at day 14, is shown below. The authors declare that these corrections do not change the results or conclusions of this paper and apologize for any inconvenience caused.

Original article: Oncotarget. 2018; 9:27667–27681. 27667-27681. https://doi.org/10.18632/oncotarget.25351

**Figure 6:**
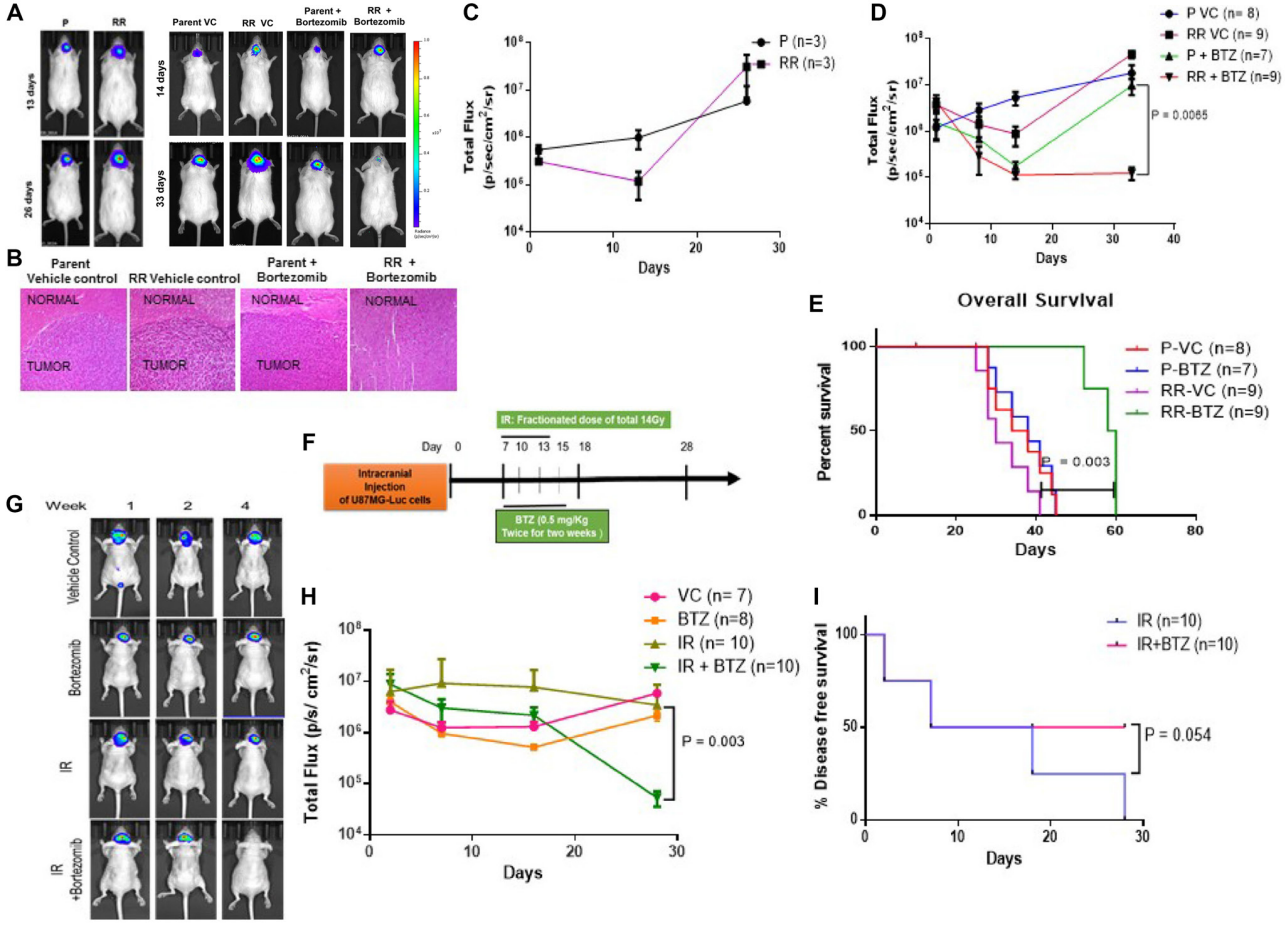
Proteasome inhibition reduces the tumorigenic potential of the cells *in vivo*. (**A**) Left panel - Representative bioluminescence images after orthotopic injection of U87MG-Luciferase labelled Parent (P) and Radiation Resistant (RR) cells. Right Panel - Bioluminescent images after orthotopic injection of U87MG-Luciferase labelled Parent (P) and Radiation Resistant (RR) cells treated with Vehicle Control (VC) and Bortezomib. (**B**) Hematoxylin and eosin (H&E) staining of mice brain slices. Brain slices of the brain tissue from mice injected with Parent Vehicle control, RR Vehicle Control, Parent + Bortezomib, RR + Bortezomib cells were formalin fixed and paraffin embedded. Sections stained with H&E show regions infiltrated with tumour cells. All photomicrographs are shown with the same magnification. Bar = 100 μm. (**C**) Graph represents bioluminescence signal at different days post injection in mice injected with P and RR cells. (**D**) Graph represents bioluminescence intensity at different days post injection of mice injected with P and RR cells pretreated with bortezomib as compared to P and RR cells treated with vehicle control. ‘n’ represents number of mice per group. (**E**) Kaplein Meier Curve for the overall survival of the mice in the pretreated study. (**F**) Schematic representation for studying the effect of intraperitoneal injections of bortezomib along with radiation treatment of mice intracranially injected with parent GBM cells. IR–Radiation; BTZ–Bortezomib. (**G**) Representative bioluminescence images of tumor formation in the mice treated with IR and BTZ compared to the mice which were administered with Vehicle Control (VC), only BTZ and only IR. (**H**) Graphical representation of bioluminescence intensity recorded for mice treated with IR and BTZ compared to the mice which were administered only saline as Vehicle Control (VC), only BTZ, only IR. (**I**) Kaplein Meier Curve for % tumor free animals in the radiation and intraperitoneally administered BTZ study.

