# Supplementary material for: Enhanced proteasomal activity is essential for long term survival and recurrence of innately radiation resistant residual glioblastoma cells
Source: Oncotarget. 2018 Jun 12;9(45):27667–81. doi: 10.18632/oncotarget.25351 (PMC6021241; doi:10.18632/oncotarget.25351)
Supplement: Supplementary file 1 [file oncotarget-09-27667-s001.pdf]

Enhanced proteasomal activity is essential for long term survival and recurrence of innately radiation resistant residual glioblastoma cells

SUPPLEMENTARY MATERIALS

GeneName	Protein Name	Relative Peptide Intensities in RR	Ub Position Glycyl lysine isopeptide	References
APP	Amyloid beta A4 protein	0.191	763	
HIST1H1B	Histone H1.5 (Histone H1a) (Histone H1b) (Histone H1s-3)	0.475	17	
HIST1H1B	Histone H1.5 (Histone H1a) (Histone H1b) (Histone H1s-3)	0.475	219	
HIST1H4A	Histone H4	0.477	13	
HIST1H4A	Histone H4	0.477	92	
KDM1A	lysine-specific histone demethylase 1A isoform b	0.478	503	Han X et al, Mol Cell. 2014 Aug
PEF1	peflin	0.508	137	McGourty CA et al, Cell. 2016 Oct
PPIA	peptidyl-prolyl cis-trans isomerase A	0.570	28	Visvikis O et al, FEBS J. 2008 Jan
RAC1	ras-related C3 botulinum toxin substrate 1 isoform Rac1	0.581	147	
RAN	GTP-binding nuclear protein Ran	0.601	71	
RBBP7	histone-binding protein RBBP7 isoform 2	0.602	4	
RBBP7	histone-binding protein RBBP7 isoform 2	0.605	159	
RPL10	60S ribosomal protein L10 isoform a	0.605	188	
RPS10	40S ribosomal protein S10	0.619	138	Sundaramoorthy E et al, Mol Cell. 2017 Feb 16
RPS10	40S ribosomal protein S10	0.626	139	Sundaramoorthy E et al, Mol Cell. 2017 Feb 16
TCEA1	transcription elongation factor A protein 1 isoform 1	0.626	55	
TDRKH	tudor and KH domain-containing protein isoform a	0.672	65	Cunningham et al, Nature Cell Biology 2015
TDRKH	tudor and KH domain-containing protein isoform a	0.672	76	Cunningham et al, Nature Cell Biology 2015
TDRKH	tudor and KH domain-containing protein isoform a	0.672	110	Cunningham et al, Nature Cell Biology 2015
TDRKH	tudor and KH domain-containing protein isoform a	0.672	112	Cunningham et al, Nature Cell Biology 2015
TDRKH	tudor and KH domain-containing protein isoform a	0.672	152	Cunningham et al, Nature Cell Biology 2015
TDRKH	tudor and KH domain-containing protein isoform a	0.672	175	Cunningham et al, Nature Cell Biology 2015
TDRKH	tudor and KH domain-containing protein isoform a	0.672	181	Cunningham et al, Nature Cell Biology 2015
TDRKH	tudor and KH domain-containing protein isoform a	0.672	187	Cunningham et al, Nature Cell Biology 2015
TDRKH	tudor and KH domain-containing protein isoform a	0.672	193	Cunningham et al, Nature Cell Biology 2015
TDRKH	tudor and KH domain-containing protein isoform a	0.672	256	Cunningham et al, Nature Cell Biology 2015
TDRKH	tudor and KH domain-containing protein isoform a	0.672	267	Cunningham et al, Nature Cell Biology 2015
TDRKH	tudor and KH domain-containing protein isoform a	0.672	479	Cunningham et al, Nature Cell Biology 2015
TDRKH	tudor and KH domain-containing protein isoform a	0.672	510	Cunningham et al, Nature Cell Biology 2015
TDRKH	tudor and KH domain-containing protein isoform a	0.672	529	Cunningham et al, Nature Cell Biology 2015
UBE2T	ubiquitin-conjugating enzyme E2 T	0.685	91	Alpi AF1 et al, Mol Cell. 2008 Dec 26
UBE2T	ubiquitin-conjugating enzyme E2 T	0.685	182	Alpi AF1 et al, Mol Cell. 2008 Dec 26

Supplementary Figure 1: Downregulated proteasome target proteins. List of downregulated proteins with ubiquitin binding lysine residues.

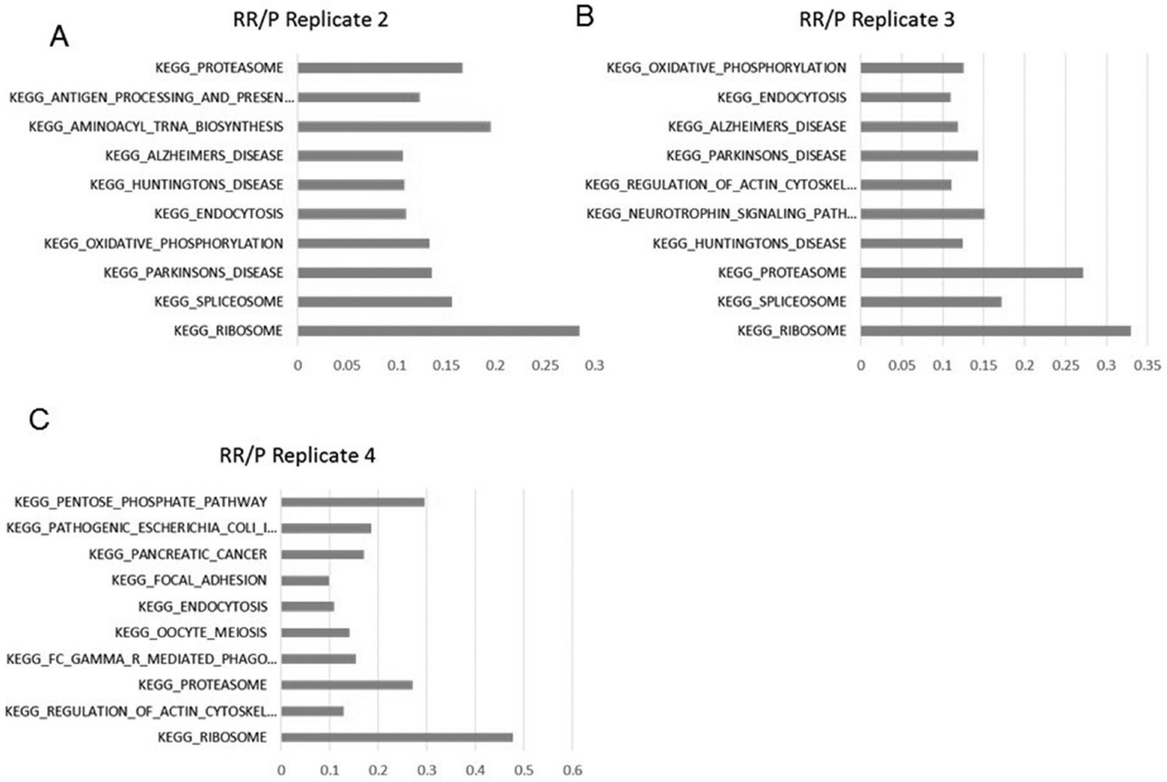

Supplementary Figure 2: Pathway analysis of deregulated proteins in all the biological replicates. (**A**) Pathway analysis of deregulated proteins in replicate 2. (**B**) Pathway analysis of deregulated proteins in replicate 3. (**C**) Pathway analysis of deregulated proteins in replicate 4.
